# Routine circumferential patellar denervation during total knee arthroplasty did not consistently eliminate neural structures at the lateral patellar border: A prospective randomised controlled trial with histological outcomes

**DOI:** 10.1002/jeo2.70800

**Published:** 2026-06-26

**Authors:** Daniel Petek, Gishane Rossanaly, Alexander Frank Heimann, Julien Hirt, Grégoire Thürig, Edouard Stauffer, Joseph M. Schwab, Marc Barrera Uso

**Affiliations:** ^1^ Department of Orthopaedic Surgery and Traumatology Hospital and University of Fribourg Fribourg Switzerland; ^2^ Promed Laboratoire Médical SA Marly Switzerland

**Keywords:** denervation, histopathology, nerve fibres, patella, total knee arthroplasty

## Abstract

**Purpose:**

Circumferential patellar denervation is widely performed during total knee arthroplasty under the assumption that electrocautery ablates nociceptive neural structures at the lateral patellar border. However, the microscopic effect of this procedure on neural substrates remains poorly defined. This study aimed to determine (1) whether histological characteristics differ between non‐denervated and denervated lateral patellar borders, and (2) which neural elements are affected by routine circumferential denervation.

**Methods:**

In this prospective randomised controlled trial with histological outcomes, 19 patients undergoing primary total knee arthroplasty were allocated to either a Facetectomy‐First Group (*n* = 10) or a denervation‐first group (*n* = 9). In the Facetectomy‐First Group, lateral patellar facetectomy was performed prior to electrocautery; in the denervation‐first group, circumferential monopolar electrocautery (~ 4 mm depth) was performed before facetectomy. Resected specimens were processed using standard histological techniques (Hematoxylin and Eosin, Masson's trichrome). Tissue compartments and neural structures, including nerve fibres and free nerve endings, were analysed semi‐quantitatively by a blinded highly experienced musculoskeletal pathologist.

**Results:**

All specimens demonstrated a consistent layered architecture with loose connective tissue present in 100% of cases. Nerve fibres were identified in connective tissue in all specimens and in most adipose layers, irrespective of surgical sequence. nerve fibres were detected in multiple tissue compartments in both groups. The mean number of nerve fibres groups per specimen was higher in the Facetectomy‐First Group (1.6 ± 0.7) than in the Denervation‐First Group (1.1 ± 0.3), without reaching statistical significance (*p* = 0.07). Although focal thermal alterations were observed in denervated specimens, morphologically identifiable nerve fibres persisted.

**Conclusions:**

Routine circumferential patellar denervation did not consistently eliminate neural structures at the lateral patellar border, and no significant histological differences were observed between the Facetectomy‐First and Denervation‐First Groups. These findings suggest that standard electrocautery‐based denervation may not reliably disrupt morphologically identifiable neural structures in this region. The functional implications of these findings remain uncertain and were not assessed in this study. Further investigations integrating advanced neural characterisation and clinical correlation are required to better define the biological and clinical impact of this procedure.

**Level of Evidence:**

Level III.

**Abbreviations**
DFG: Denervation‐First Group
FFG: Facetectomy‐First Group
FNEs: free nerve endings
IQR: interquartile range
SD: standard deviation
TKA: total knee arthroplasty

## INTRODUCTION

Knee osteoarthritis is among the most prevalent degenerative joint diseases worldwide and a major cause of pain and disability [13]. In patients with persistent symptoms despite conservative treatment, total knee arthroplasty (TKA) is widely performed and provides reliable pain relief and functional improvement [14, 21]. Nevertheless, postoperative anterior knee pain remains a frequent and clinically relevant issue after TKA [16]. To address this problem, several patella‐related strategies have been proposed, including patellar resurfacing, lateral patellar facetectomy and patellar denervation [12, 27].

Patellar resurfacing is performed selectively; however, due to concerns about complications such as periprosthetic fractures and the potential need for revision surgery, lateral patellar facetectomy has been proposed as a validated alternative [9, 18, 28]. This procedure aims to reduce anterior knee pain by addressing lateral patellofemoral mechanical conflict while preserving patellar biomechanics and overall joint stability [12]. Patellar denervation is another commonly used adjunctive technique, either alone or in combination with facetectomy or resurfacing [12].

Despite its widespread use and favourable safety profile, the clinical benefit of patellar denervation remains uncertain. Several clinical studies [4, 5, 25, 29] have failed to demonstrate a consistent reduction in postoperative anterior knee pain compared with standard TKA techniques [22].

Despite the extensive clinical literature [7, 15, 19, 21, 29], the actual effect of electrocautery on neural structures at the lateral patellar border remains poorly understood [12]. It is unclear whether routine circumferential denervation effectively disrupts peripatellar neural structures. Most available studies focus on clinical outcomes, with limited histological evidence supporting the presumed mechanism of action [15, 25, 31].

In this context, a better understanding of the structural effect of electrocautery on peripatellar neural tissues is essential, as the biological mechanism underlying patellar denervation has not been directly validated at the histological level. Importantly, establishing whether neural structures persist after routine denervation may help explain the inconsistent clinical findings reported in the literature and clarify the mechanistic relevance of this widely used technique.

We therefore asked: (1) How does the histologic analysis of non‐denervated lateral patellar borders differ from that of denervated patellar borders; and (2) what is the degree of neural structure involvement following routine patellar denervation, as assessed by histologic analysis of lateral patellar facetectomy specimens obtained during primary TKA?

We hypothesised that routine patellar denervation would not result in a consistent or substantial reduction in neural structure involvement at the lateral patellar border.

## MATERIALS AND METHODS

### Study design and patient selection

This single‐centre, prospective randomised controlled trial with histological outcomes was conducted at the University‐Hospital of Fribourg, Switzerland, between June and September 2023. The study protocol was approved by the regional ethics committee (CER‐VD ID 2022‐02313) and complied with the principles of the Declaration of Helsinki. All participants provided written informed consent prior to enrolment.

Consecutive patients scheduled for primary TKA were screened for eligibility. Patients were eligible if they were adults (> 18 years) undergoing primary TKA and had provided signed informed consent. Patients were excluded in cases of refusal or inability to provide informed consent, prior knee surgery, history of patellar fracture, systemic infection or previous septic arthritis of the knee, as well as the presence of intraoperative macroscopic signs of joint infection.

After exclusion of one patient due to prior knee surgery, 19 patients (19 knees) were included in the final analysis. Randomisation was performed using a computer‐generated sequence by an independent investigator. Due to the nature of the surgical intervention, allocation concealment was not feasible, and the operating surgeon was aware of group assignment at the time of surgery. Participants were allocated into two groups, based on the timing of lateral patellar border specimen harvesting relative to patellar denervation.

### Group definitions and surgical sequence

**Figure 1:**
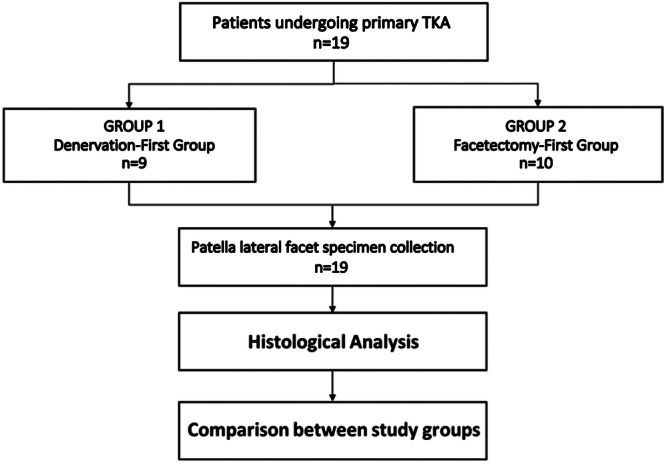
Depiction of the study procedure. TKA, total knee arthroplasty.

Two groups were defined: (Figure 1)

- Denervation‐First Group (DFG): Circumferential patellar denervation was performed first using monopolar electrocautery applied circumferentially along the peripatellar soft tissues at the lateral patellar border. A partial lateral patellar facetectomy was then carried out, and the resected specimen was harvested for histological analysis.
- Facetectomy‐First Group (FFG): A partial lateral patellar facetectomy was performed first. The resected lateral patellar border specimen was harvested immediately before any electrocautery. Circumferential patellar denervation was then performed using the same standardised technique.

In both groups, the surgical techniques for facetectomy and denervation were identical. The only difference was the sequence of specimen harvesting relative to electrocautery.

### Surgical technique

**Figure 2:**
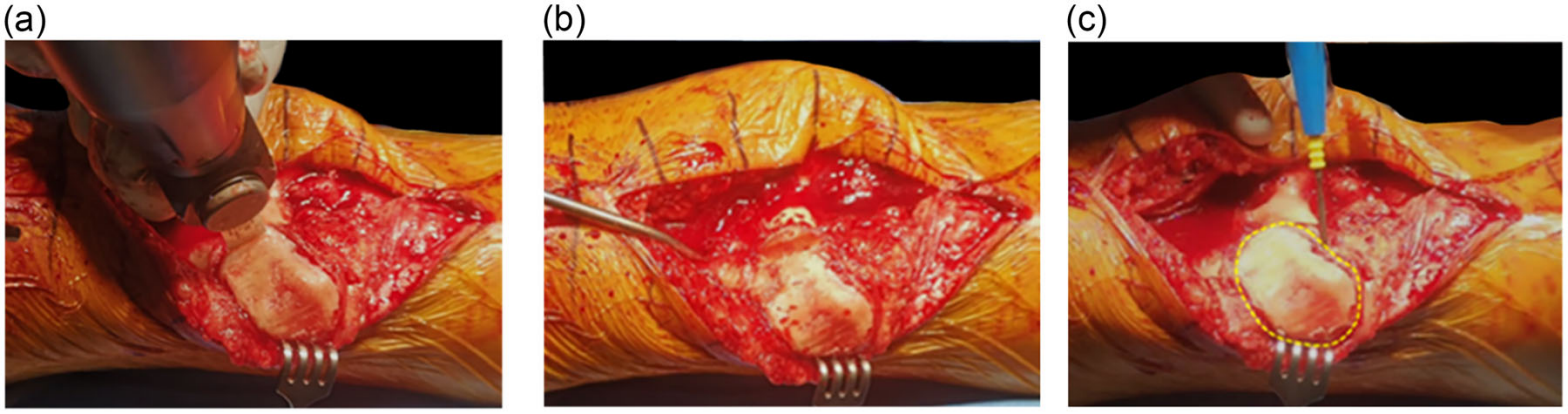
Intraoperative images showing (a) an inverted right patella with the oscillating saw positioned on the lateral patellar border; (b) the articular surface after partial lateral patellar facetectomy; (c) circumferential patellar denervation (dashed yellow line).

All procedures were performed via a standard medial parapatellar approach (Figure 2). The patella was systematically everted after medial parapatellar arthrotomy and prior to any bone preparation to ensure consistent surgical handling [20].

**Figure 3:**
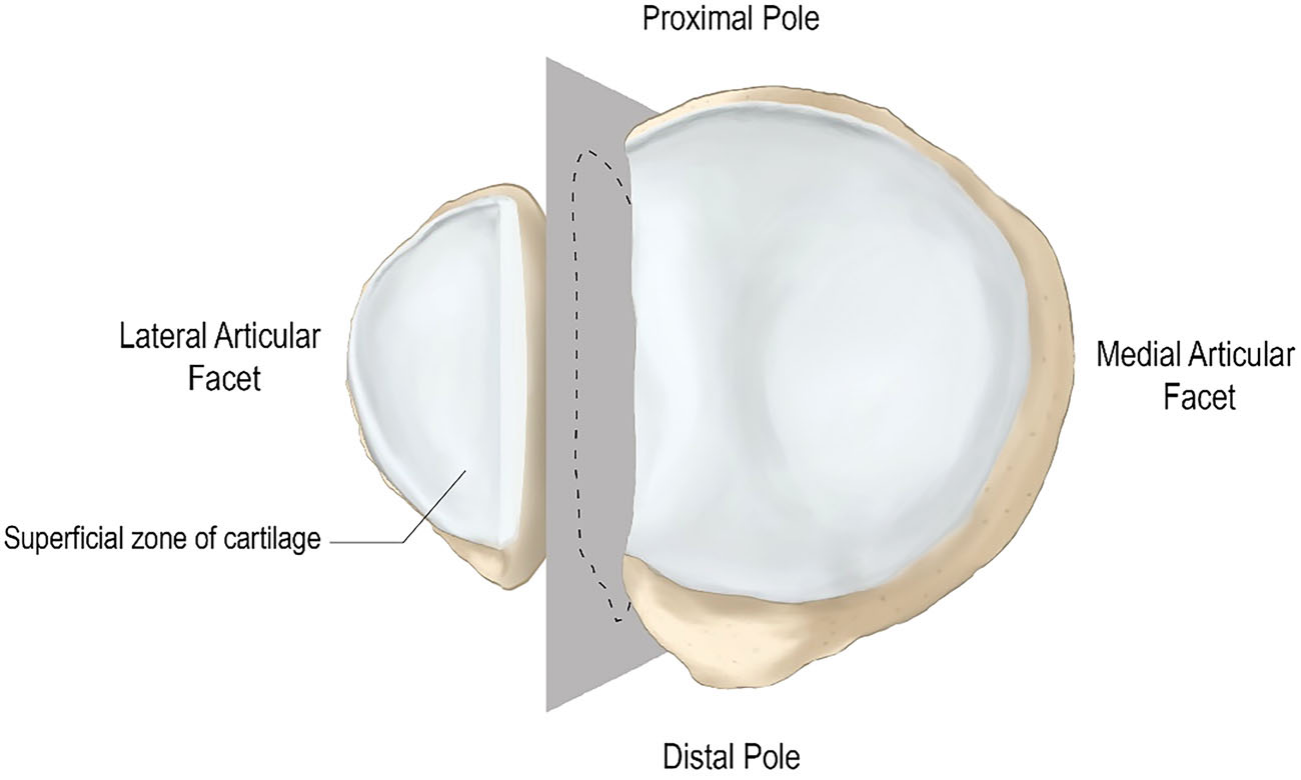
Articular surface view of left lateral patellar facetectomy following patellar eversion through a standard medial parapatellar approach, with resection of the lateral patellar facet performed using an oscillating saw and completed with a scalpel (*illustration by Johan Duchamp*).

Partial lateral patellar facetectomy was performed under direct visualisation using an oscillating saw. The resection was limited to the lateral border of the patella and oriented along the plane between the anterior patellar surface and the posterior aspect of the patellar tendon. Care was taken to preserve the patellar tendon and adjacent extensor structures. Residual soft tissue attachments were released sharply to remove the entire facet. The fragment was released using a cold scalpel to avoid additional thermal damage to surrounding tissues (Figure 3).

Circumferential patellar denervation was performed circumferentially to the peripatellar soft tissues, avoiding penetration into the patellar bone (depth ~4 mm). Electrocautery was performed using a standard monopolar device under routine clinical settings. Specific parameters such as power output and application duration were not formally standardised or recorded. All procedures were performed by a single senior knee orthopaedic surgeon experienced in TKA, following a standardised surgical technique.

All patients underwent both facetectomy and denervation. Group assignment only determined the timing of specimen collection. For histological evaluation, the entire resected lateral patellar border including cortical bone, subchondral bone, and attached soft tissues was collected. Specimen dimensions were approximately 2–3 cm in width × 1–1.5 cm in length × 1–1.5 cm in thickness. Samples were immediately fixed in 4% buffered formalin and sent for histological analysis.

Subsequent TKA steps (tibial and femoral cuts, trialling and component implantation) followed standard institutional procedures. Patellar resurfacing was left to the discretion of the senior surgeon and was not a study variable.

### Histological processing and analysis

Neural structures were identified using standard histological criteria based on morphological features observable with conventional staining. Nerve fibres were defined as organised bundles of neural elements.

All specimens were processed at our senior pathologist's lab Fixation was performed in 4% buffered formalin for ≥ 24 h, followed by controlled EDTA decalcification, dehydration, and paraffin embedding. Serial 2 µm sections were stained with hematoxylin‐eosin (H&E) and Masson's trichrome.

**Figure 4:**
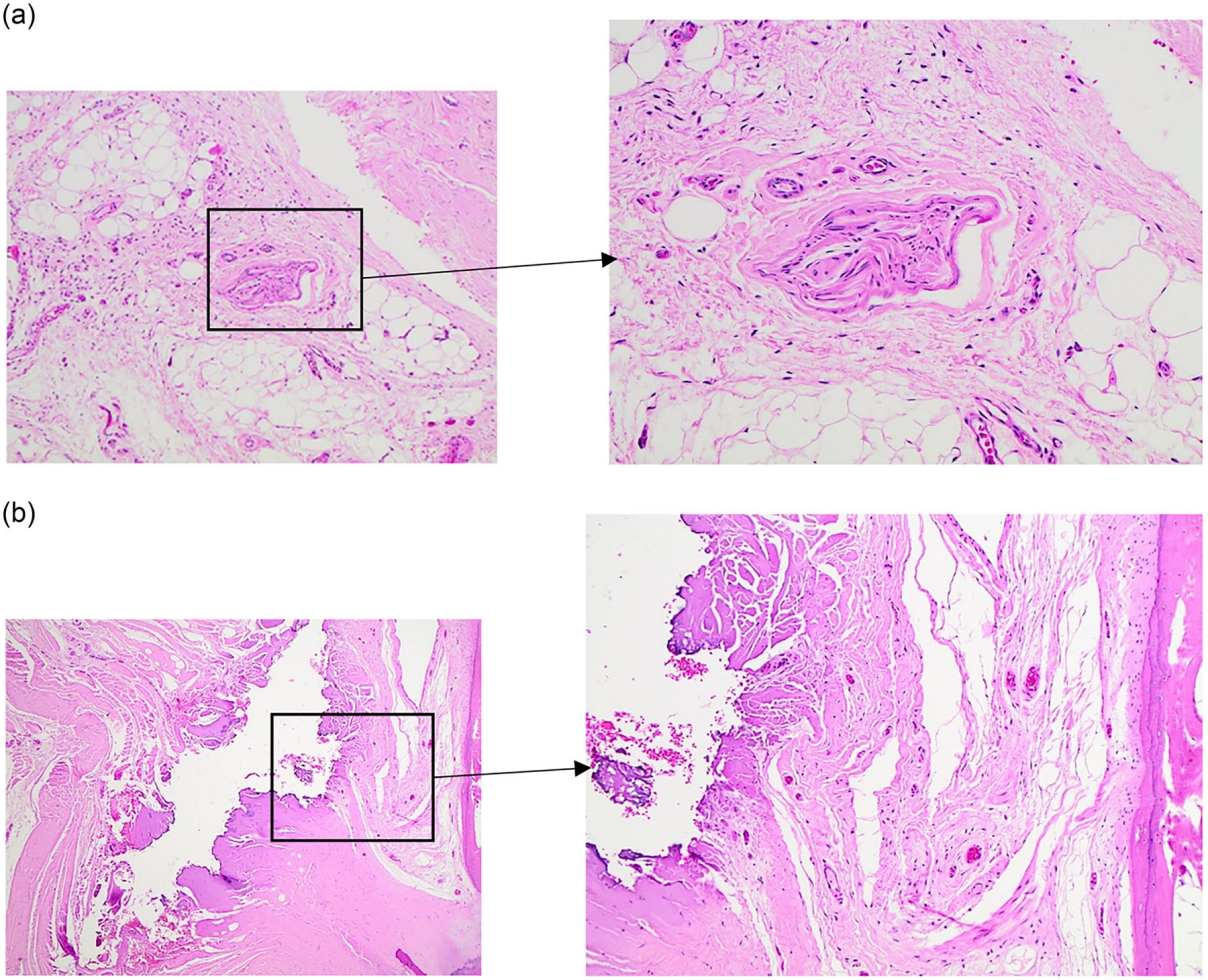
(a) FFG shows a non‐denervated specimen with a well‐preserved nerve fibre exhibiting organised fascicular structure and clear boundaries, without evidence of cauterisation damage. (b) DFG shows a denervated specimen stained with hematoxylin–eosin. A nerve fibre within loose connective tissue displays morphological changes including irregular contours and partial fragmentation, with adjacent thermal tissue alterations. DFG, Denervation‐First Group; FFG, Facetectomy‐First Group.

Slides were evaluated by an experienced musculoskeletal pathologist blinded to group allocation. The assessment included identification of tissue layers (connective, adipose, synovial, cartilage and bone), as well as evaluation of the presence, location, and density of nerve fibres, including free nerve endings (FNEs), and the depth and extent of thermal tissue changes (Figure 4a,b). The analysis of tissue compartments was performed to provide anatomical context for neural structure localisation, given the heterogeneous distribution of nerve fibres across peripatellar tissue layers.

In accordance with the framework described by Biedert et al. [3], nerve fibres and FNEs were assessed semi‐quantitatively across the full thickness of the specimens. All nerve structures were classified according to size, based on the number of fibres per fascicle (Table 1). These assessments were performed by a senior musculoskeletal pathologist with prior experience in neural structure analysis, including previous collaboration with Biedert.

**Table 1:** Semi‐quantitative grading of nerve fibres and free nerve endings adapted from Biedert et al. [18].

Parameter	Category	Definition
Detectability	Significant searching	Nerve fibres and FNEs identified only after extensive targeted examination
	Minor searching	Nerve fibres and FNEs identified after limited directed examination
	Evident	Nerve fibres and FNEs readily visible without targeted search
Quantity per bundle	<5 fibres	Small nerve bundles with fewer than five fibres
	>5 fibres	Larger nerve bundles with more than five fibres
Distribution	Superficial	Located in superficial soft tissue layers
	Deep	Located in deeper tissue layers close to bone or cartilage

Abbreviation: FNEs, free nerve endings.

This approach was selected to allow a consistent and reproducible comparison between specimens, while accounting for the inherent variability in tissue sampling and sectioning. Given the exploratory nature of the study and the absence of immunohistochemical staining, a pragmatic morphological classification was considered appropriate. A semi‐quantitative threshold (< 5 vs. ≥ 5 nerve fibres per bundle) was used to differentiate low‐density from higher‐density neural structures across specimens. This approach was selected in the absence of established histological classifications for nerve fibre quantification in this context and was applied consistently across all samples. This threshold is exploratory and does not represent a validated biological cutoff.

### Statistical analysis

Given the exploratory and descriptive nature of this study, no a priori power analysis was performed. Continuous variables are reported as median with interquartile range (IQR) or as mean ± standard deviation (SD), depending on their distribution, while categorical variables are presented as absolute numbers and percentages.

Comparisons between the FFG and the DFG were performed according to the type of data analysed. Continuous demographic variables, such as age, were compared using the Mann–Whitney *U* test due to the small sample size and the non‐parametric distribution of the data. Categorical variables, including sex, side distribution, tissue compartment presence and nerve fibre presence, were analysed using the two‐sided Fisher's exact test.

For the semi‐quantitative assessment of nerve fibres, the proportion of specimens with ≥ 5 versus < 5 nerve fibres were compared between groups using a Fisher's test of proportions. In addition, the mean number of nerve fibre groups per specimen was compared using a two‐sided unpaired Student's t‐test.

All statistical tests were two‐sided, and a *p*‐value < 0.05 was considered statistically significant. Statistical analyses were performed using MedCalc and R software.

## RESULTS

### Patient characteristics

Nineteen patients (19 knees) were included in the analysis: 10 in the FFG and 9 in the DFG. Median age was 69 years (IQR 64–74) in the FFG and 70 years (IQR 65–78) in the DFG with no statistically significant difference (*p*‐value = 0.44). In the FFG, three patients were male (30%) and seven were female (70%), whereas in the DFG, four patients were male (44%) and five were female (56%). Side distribution (right vs. left knee) and sex ratio were also comparable between groups (Table 2).

**Table 2:** Demographic characteristics of the study population.

Variable	Facetecomy‐First Group (*n* = 10)	Denervation‐First Group (*n* = 9)	*p*‐Value
Age (years), median (IQR)	69 (64–74)	70 (65–78)	0.44
Side, *n* (%)			1.00
Right	6 (60%)	5 (56%)	
Left	4 (40%)	4 (44%)	
Sex, *n* (%)			0.65
Male	3 (30%)	4 (44%)	
Female	7 (70%)	5 (56%)	

Abbreviation: IQR, interquartile range.

### Histological composition of resected specimens

All harvested specimens were suitable for histological evaluation. Loose connective tissue and cartilage were present in all specimens in both groups (FFG: 10/10; DFG: 9/9) adipose tissue was observed in 7/10 FFG specimens (70.0%) and 9/9 DFG specimens (100%), with no statistically significant difference (*p* = 0.21). Synovial tissue was present in 7/9 FFG specimens (77.8%) and 8/9 DFG specimens (88.9%) (Table 3).

**Table 3:** Distribution of histological tissue compartments and associated nerve fibre presence identified in lateral patellar border specimens from both groups.

Tissue compartment and nerve presence	Facetectomy‐First Group, *n* (%)	Denervation‐First Group, *n* (%)	*p*‐Value (Fisher's exact test)
Loose connective tissue	10/10 (100%)	9/9 (100%)	1
Adipose tissue	7/10 (70.0%)	9/9 (100%)	0.21
Synovial tissue	3/7 (42.9%)	2/8 (25%)	0.61

*Note*: Denominators reflect only the number of specimens in which the corresponding tissue layer was present.

### Presence of nerve fibres

Nerve fibres were consistently identified within loose connective tissue in all specimens in both groups. When adipose tissue was present, nerve fibres were observed in almost all cases in both groups (FFG: 7/7; DFG: 8/9; *p* = 1.00). Within synovial tissue, nerve fibres were observed in 3/7 FFG specimens (42.9%) and 2/8 DFG specimens (25.0%) (*p* = 0.61).

### Semi‐quantitative assessment of nerve fibres and FNEs

Nerve fibres and FNEs were identified in both groups across all tissue compartments (Table 4 **).**

**Table 4:** Semi‐quantitative assessment of nerve fibres across all available soft‐tissue layers.

	Facetectomy‐First Group	Denervation‐First Group	*p*‐Value*
Significant searching			
≥5 nerve fibres	0/10 (0%)	0/9 (0%)	NA
<5 nerve fibres	5/10 (50%)	2/9 (22%)	*p* = 0.35
Minor Searching			
≥5 nerve fibres	2/10 (20%)	2/9 (22%)	*p* = 1
<5 nerve fibres	3/10 (30%)	0/9 (0%)	*p* = 0.21
Evident			
≥5 nerve fibres	5/10 (50%)	3/9 (33%)	*p* = 0.64
<5 nerve fibres	1/10 (10%)	3/9 (33%)	*p* = 0.30
Total			
≥5 nerve fibres	7/30 (23%)	5/27 (18.5%)	*p* = 0.75
<5 nerve fibres	9/30 (30%)	5/27 (18.5%)	*p* = 0.37
Nerve fibres groups per specimen (mean ± SD)	1.6 ± 0.7	1.1 ± 0.3	*p* ^+^ = 0.07

* Calculated from Fisher's exact test. ^+^Calculated from two‐sided T‐test.

Overall, the mean number of nerve fibres and FNE groups per specimen was higher in the FFG (1.6 ± 0.7) compared to the DFG (1.1 ± 0.3), but this difference did not reach statistical significance (*p* = 0.07).

## DISCUSSION

The main finding of the present histological study was that circumferential patellar denervation did not consistently eliminate neural structures at the lateral patellar border, and the composition of neural structures was largely similar between specimens harvested before and after circumferential patellar denervation. Neural structures, including nerve fibres and FNEs, were observed across multiple tissue compartments regardless of the surgical sequence. These findings may suggest that routine electrocautery‐based denervation may not reliably disrupt peripatellar nociceptive pathways and may therefore have limited clinical relevance.

In addressing the first study question, the histological composition of the superior, inferior, and lateral patellar border was comparable between groups. Both the Facetectomy‐First and Denervation‐First specimens demonstrated a consistent layered architecture composed of loose connective tissue, adipose tissue, cartilage, and variably present synovial tissue. Neural structures were identified across multiple tissue compartments in both groups, without significant differences in their presence or distribution. These findings suggest that circumferential electrocautery, as routinely performed, does not markedly alter the overall histological organisation or the spatial distribution of neural elements in this region [2, 3, 7, 10, 11, 17, 19, 23, 26, 30, 31, 32].

Regarding the second study question, neural structures, including nerve fibres and FNEs, remained consistently identifiable following electrocautery. Although a non‐significant trend toward fewer nerve fibre groups per specimen was observed in the DFG, this difference did not reach statistical significance and should be interpreted cautiously given the limited sample size.

Taken together, these results suggest that routine patellar denervation may exert, at most, a partial and inconsistent effect on morphologically identifiable neural structures. An important consideration is whether circumferential electrocautery provides any incremental biological effect beyond the tissue disruption inherent to lateral patellar facetectomy itself. Surgical exposure and resection of the lateral patellar facet necessarily involve incision and removal of peri‐patellar soft tissues, which may already interrupt a proportion of neural structures. In this context, our findings suggest that additional circumferential electrocauterization may not provide a meaningful incremental biological effect, as neural elements remained consistently detectable regardless of the sequence of procedures. This raises the possibility that the biological impact of routine patellar denervation may be limited and, at least in part, redundant with the mechanical effects of facetectomy [9, 12, 20, 27, 28].

From a mechanistic perspective, several factors may explain the persistence of neural structures following electrocautery. First, neural elements were distributed across multiple tissue layers, including deeper connective and adipose tissues, which may be variably affected by thermal energy. Second, the depth and uniformity of electrocautery applications are inherently operator‐dependent and may not consistently reach all neural structures. Third, thermal diffusion may result in heterogeneous tissue effects, with superficial coagulation and relative preservation of deeper fibres. These factors collectively suggest that routine electrocautery may not achieve complete structural disruption of peripatellar innervation [1, 2, 3, 8, 17, 19, 23, 25, 26, 31].

The clinical relevance of these findings should be interpreted considering the existing literature, which has reported inconsistent or limited benefits of patellar denervation in TKA. While some studies and meta‐analyses have suggested a reduction in anterior knee pain during early follow‐up, this effect appears inconsistent and often diminishes over time [4, 5, 15, 22, 25, 28, 29]. Rather than contradicting these observations, the present findings provide a potential mechanistic explanation for them. The persistence of neural structures observed histologically suggests that routine electrocautery may not reliably eliminate all morphologically identifiable neural pathways at the lateral patellar border, thereby contributing to the variability and often limited clinical impact reported in prior studies [5, 6, 7, 12, 15].

Previous anatomical and histological studies have demonstrated that the patella is innervated by branches of the medial and lateral patellar nerves, with neural elements and nociception‐related fibres identified in the peripatellar soft tissues [2, 3, 17, 19, 23, 26, 31]. More recent work has also highlighted the complexity and variability of anterior knee innervation, suggesting that circumferential electroablation may be an anatomically imprecise means of targeting pain‐generating neural structures [24]. Our findings are consistent with this anatomical complexity, as neural elements were observed across multiple tissue compartments rather than confined to a superficial layer. This distribution further supports the hypothesis that a uniform and complete denervation through surface electrocautery may be difficult to achieve.

The present study has several limitations. First, the sample size was limited, reducing the statistical power to detect subtle differences between groups and rendering the observed trends exploratory. Second, the analysis was restricted to the lateral, superior, and inferior patellar border and cannot be extrapolated to the entire patella or the global patellofemoral innervation. Third, the study provides purely morphological data without assessment of neural functionality or correlation with clinical outcomes such as anterior knee pain. Fourth, although conventional histological staining allowed reliable identification of larger nerve fibres and overall structural assessment, the absence of immunohistochemical techniques represents a limitation. Such methods may offer greater sensitivity for detecting smaller or specific neural subtypes, and their absence may have limited the ability to fully characterise neural elements [3, 24, 30]. In addition, the absence of an a priori sample size calculation limits the ability to exclude a type II error, and the study may be underpowered to detect subtle between‐group differences. Finally, all procedures were performed in a single institution by the same surgical team, which may limit generalisability.

This study was designed as a structural histological investigation focusing on morphologically identifiable neural elements following routine clinical electrocautery. Future studies incorporating immunohistochemical techniques as well as correlation with clinical outcomes, are warranted to better define the functional and clinical significance of these findings.

## CONCLUSION

Routine circumferential patellar denervation did not consistently eliminate neural structures at the lateral patellar border, and no significant histological differences were observed between the Facetectomy‐First and Denervation‐First groups. These findings suggest that standard electrocautery‐based denervation may not reliably disrupt morphologically identifiable neural structures in this region. The functional implications of these findings remain uncertain and were not assessed in this study. Further investigations integrating advanced neural characterisation and clinical correlation are required to better define the biological and clinical impact of this procedure.

## AUTHOR CONTRIBUTIONS

**Daniel Petek**: Study conception and design, surgical procedures, supervision of the project, critical revision of the manuscript. **Gishane Rossanaly**: Study design, specimen collection, data acquisition, statistical analysis, drafting of the manuscript, and manuscript revision. **Alexander Frank Heimann**: Surgical procedures, patient recruitment. **Julien Hirt**: Data collection, critical revision of the manuscript. **Grégoire Thürig**: Surgical procedures, data acquisition, critical revision of the manuscript. **Edouard Stauffer**: Histological processing and analysis, interpretation of histological findings, contribution to the methodology section, critical revision of the manuscript. **Joseph M. Schwab**: Study supervision, statistical analysis, critical revision of the manuscript. **Marc Barrera Uso**: Study conception and design, surgical procedures, critical revision of the manuscript. All authors read and approved the final manuscript.

## FUNDING

The authors have no funding to report.

## CONFLICT OF INTEREST STATEMENT

The authors declare no conflicts of interest.

## ETHICS STATEMENT

Please include the name of the institutional review board (IRB) and the approval number. If not applicable, please state so.: The study protocol was approved by the regional ethics committee (CER‐VD ID 2022‐02313) and complied with the principles of the Declaration of Helsinki. All patients provided written informed consent prior to participation in this study.

## Data Availability

The data that support the findings of this study are available from the corresponding author upon reasonable request. The data sets generated and analysed during the current study are not publicly available due to institutional data protection regulations and patient confidentiality requirements.
